# Are Animals a Neglected Transmission Route of SARS-CoV-2?

**DOI:** 10.3390/pathogens9060480

**Published:** 2020-06-18

**Authors:** Marta Hernández, David Abad, José María Eiros, David Rodríguez-Lázaro

**Affiliations:** 1 Microbiology Division, Faculty of Science, University of Burgos, Plaza Misael Bañuelos s/n, 09001 Burgos, Spain; hernandez.marta@gmail.com (M.H.); dabad87@gmail.com (D.A.); 2 Clinical Microbiology Department, Hospital Universitario del Río Hortega, C/Dulzaina 2, 47012 Valladolid, Spain; jmeirosbouza@gmail.com

**Keywords:** SARS-CoV-2, COVID-19, Food Safety, One Health, epidemiology, neglected route

## Abstract

Little information on the SARS-CoV-2 virus in animals is available to date. Whereas no one husbandry animal case has been reported to date, which would have significant implications in food safety, companion animals play a role in COVID-19 epidemiology that opens up new questions. There is evidence that SARS-CoV-2 can infect felines, dogs and minks, and there is evidence of human-to-animal infection. Likewise, the S protein nucleotide sequence of the SARS-CoV-2 virus isolated in domestic animals and humans is identical, and the replication of the SARS-CoV-2 in cats is efficient. Besides, the epidemiological evidence for this current pandemic indicates that the spillover to humans was associated with close contact between man and exotic animals, very probably in Chinese wet markets, thus there is a growing general consensus that the exotic animal markets, should be strictly regulated. The examination of these findings and the particular role of animals in COVID-19 should be carefully analyzed in order to establish preparation and containment measures. Animal management and epidemiological surveillance must be also considered for COVID-19 control, and it can open up new questions regarding COVID-19 epidemiology and the role that animals play in it.

Most coronaviruses affecting humans have a common origin in different species of bats. Different species of mammals act as an intermediate host. In the case of SARS-CoV-2, several mammals are speculated to be intermediate hosts, including the pangolin, in which a coronavirus strain that exhibits strong similarity to SARS-CoV-2 in the receptor-binding domain (RBD) has been identified [1], which mediates virus attachment to host target cells. However, little information on the SARS-CoV-2 virus in animals is available to date, and only scarce sporadic cases have been reported by the World Organisation for Animal Health (OIE) [2]. The first reported animal case occurred on 26 February in Hong Kong; a dog whose owner was hospitalised due to COVID-19 infection tested positive for SARS-CoV-2, and remained positive up to 9 March although the animal did not show any specific clinical signs [2]. Similarly, two dogs whose owner was hospitalised due to COVID-19 infection were placed under quarantine, and one tested positive for SARS-CoV-2 on 18 March and remained positive up to 20 March (the virus was isolated from it), but again, no clinical signs were detected during the quarantine period [2]. On 27 March, a tiger (Panthera tigris) was confirmed positive for SARS-CoV-2 at the Bronx Zoo (New York, USA), and three other tigers and three lions showed clinical signs [2]. One of the three lions was confirmed positive for SARS-CoV-2 on 15 April. It was assumed that they had become infected via an asymptomatic zoo employee. A cat kept in the same household as a confirmed COVID-19 patient in Hong Kong was confirmed with SARS-CoV-2 on 30 March; nasal, oral, and rectal swab samples tested positive up to 1 April, although the cat did not exhibit any specific clinical signs [2]. On 27 March, a pet German Shepherd dog from a household with COVID-19 affected inhabitants in Richmond, New York, was sampled for respiratory illness and tested RTqPCR-positive for SARS-CoV-2 up to 21 May [2]. Two other cats from separate households in New York (Nassau and Orange Counties) were confirmed for SARS-CoV-2 by molecular testing (RTqPCR and sequencing) in mid-April [2]. Since then, other cases have been reported in other countries such as Belgium, the Netherlands, France, Germany, Russia, and Spain, affecting different domestic animals or mink farms. These reported episodes highlight that while felines and dogs can be infected by SARS-CoV-2, only felines can show clinical signs. However, it remains unclear if any domestic or livestock species can spread the virus to humans. Similarly, the susceptibility of ferrets and different domestic animals to SARS-CoV-2 has also been demonstrated in experimental infections: SARS-CoV-2 replicates poorly in dogs, pigs, chickens, and ducks, but efficiently in ferrets and cats and can be transmitted between cats via respiratory droplets [3].

A relevant role of the host receptor coding for angiotensin-converting enzyme 2 (ACE2) in COVID-19 pathogenesis has been shown and the specificity of the interaction between virus and receptor determines host tropism and range [4]. While ACE2 receptor amino acid sequences in different animals show phylogenetic distance with respect to the human ACE2 receptor, the pangolin, cat, feline, and dog ACE2 receptor sequences cluster closely (Figure 1), and it predicts that the S protein of SARS-CoV-2 may bind to ACE2 in domestic cats and dogs, as well as a range of other species, including pigs, cows, pangolins, and Chinese hamsters [4,5].

For indepth understanding of virus–host interaction at a cellular level, we compared the S protein nucleotide sequences from the SARS-CoV-2 isolated in animals with that from the virus isolated in humans, and while the S protein nucleotide sequence was different in bats and pangolins, the sequence was practically identical in the case of the dog (1 unique mismatch, i.e., >99.99% similarity), and the 6 six binding sites of the RBD of SARS-CoV-2 from other animals (cat, mink and tiger) were also identical (Figure 2).

The relationship between humans, animals (domestic, livestock, and wild) and the environment is a complex, dependent relationship in a fragile and dynamic balance. In this context, therefore, health must be considered as a global concept that not only encompasses healthcare aspects of human medicine but goes further and considers interactions with animals (animal health) and the environment (environmental health). With this premise, a few years ago, the “One Health” initiative emerged to establish cross-sectoral collaboration to guarantee the understanding and management of public health risks at the human–animal–environment interface and improve global health security [6]. In this scenario, the animal–human interaction could have affected the COVID-19 epidemiology and pathogenesis in two very different ways. Firstly, one of the most complex questions on the COVID-19 pandemic is the substantial differences in the severity observed in the Hubei province (China) and other geographical areas. One possible explanation could be the antibody-dependent enhancement (ADE) of SARS-CoV-2 due to prior exposure to other coronaviruses [7,8]. ADE modulates the immune response and can trigger violent immune reactions such as the cytokine storm, directly related to severe COVID-19 cases and deaths [7]. This phenomenon requires prior exposure to similar antigenic epitopes, and some animal species, such as dogs, pigs, and bovines, are often infected by taxonomically related coronaviruses [9,10]. Consequently, past interrelation with animals previously infected with animal coronaviruses might have primed the human immune system against them and, in turn, conferred a partial protection against the circulating SARS-CoV-2 [8,9,10], this being a possible explanation for the observed geographic limitation of severe cases and deaths.

Secondly, if human-to-animal transmission is confirmed, we need to face three scenarios in which zoonotic transmission can be considered under a One Health perspective. Firstly, livestock for human consumption. Not one husbandry animal case has been reported to date, notwithstanding geographically wide SARS-CoV-2 distribution. This is a very significant aspect of food safety since this evidence supports that animal-derived foods can be considered as low infectious risk and a negligible route of infection to humans [11]. Secondly, companion animals. This second scenario opens up new questions regarding COVID-19 epidemiology and the role that animals can play in it. There is evidence that SARS-CoV-2 can infect felines, particularly cats, and dogs [12], and there is evidence of human-to-animal infection [13]. Fifteen out of 102 (14.7%) cat sera collected after the outbreak in Wuhan city (China) tested positive using an inhouse indirect ELISA, and three also showed high virus-neutralising antibody titres [14]. The owners of these three cats have been confirmed as COVID-19 positive. Likewise, the S protein nucleotide sequence of the SARS-CoV-2 virus isolated in some domestic animals, such as dogs and cats, and humans is almost identical, particularly the six binding sites of the RBD of SARS-CoV-2 to the human ACE receptor (Figure 2), and the replication of the SARS-CoV-2 in cats is efficient [3]. SARS-CoV-2 has been found in the intestinal tract of different animal models [15], and there is evidence that human and animal guts can support its replication [16,17,18]. Given the close contact between human and domestic animals, faecal–oral transmission should not be ruled out and should be considered in COVID-19 epidemiology. These findings open up debate on considering domestic animals as efficient SARS-CoV-2 reservoirs and on the need to evaluate this transmission route for the containment of the infection, particularly in situations where the population is confined to their homes, and on whether epidemiological surveillance should be considered. Finally, the third scenario considers exotic animals, which include a large group of species. In some regions, exotic animals are used for food. The epidemiological evidence for this current pandemic indicates that the spillover to humans was associated with close contact between man and exotic animals, very probably in Chinese wet markets. Similar situations were already associated with the other two coronavirus international epidemics—SARS (civets) and MERS (camelids)—and even in epidemics in which the route of transmission was not respiratory, such as Nipah (fruit bats or pigs) or Ebola (close contact with bush animals and their blood during butchering). Consequently, there is a growing general consensus that the exotic animal markets, particularly those selling live animals, should be strictly regulated, and access to these types of animals should be limited (e.g., hunted bush animals) [19,20].

The examination of the above scenarios and the particular role of animals in COVID-19 should be carefully analyzed in order to establish preparation and containment measures. Therefore, further research studies in animals must be carried out to better understand the risks and consequences of the SARS-CoV-2 infection, to determine the role of animals as reservoirs and transmitters of SARS-CoV-2, as well as to understand the host factors involved in coronavirus replication. Animal management and epidemiological surveillance must also be considered for COVID-19 control, and it can open up new questions regarding COVID-19 epidemiology and the role that animals play in it.

## Figures and Tables

**Figure 1:**
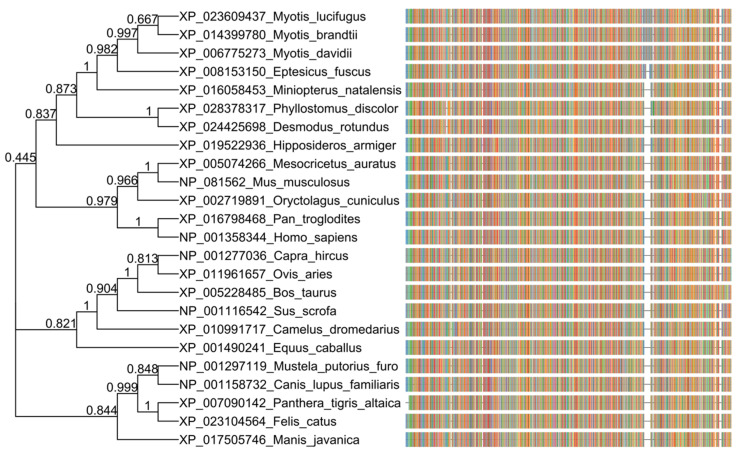
Phylogenetic analysis of amino acid sequences of the angiotensin-converting enzyme 2 (ACE2) receptor in different animals and humans. The ACE2 orthologous amino acid sequences were downloaded from NCBI (https://www.ncbi.nlm.nih.gov/gene/59272/ortholog/?scope=33554) and aligned with COBALT (https://www.ncbi.nlm.nih.gov/tools/cobalt/re_cobalt.cgi). The tree was generated using a maximum likelihood estimate with FastTree, under a JTT model. The graphic representation was made with the ggtree package in R, and each color in the multiple sequence aligment (msa) corresponds to an amino acid.

**Figure 2:**
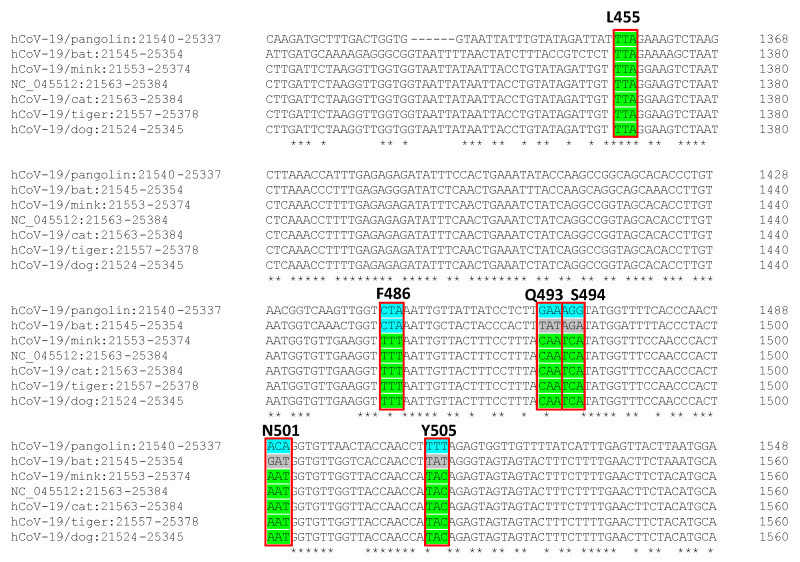
Nucleotide comparison of SARS-CoV2 spike protein from viruses isolated from animals (pangolin, bat, cat, tiger, mink, and dog) and the reference human virus isolated in Wuhan. In green, the consensus with the human SARS-CoV-2 virus sequence. Red box signs, the 6 binding sites of the receptor-binding domain (RBD) of SARS-CoV-2 to ACE receptor.
